# App-supported sleep coaching: implications for sleep duration and sleep quality

**DOI:** 10.3389/frsle.2023.1156844

**Published:** 2023-06-26

**Authors:** Suzanne B. Gorovoy, Rebecca L. Campbell, Rina S. Fox, Michael A. Grandner

**Affiliations:** ^1^Sleep and Health Research Program, Department of Psychiatry, University of Arizona College of Medicine, Tucson, AZ, United States; ^2^Biobehavioral Health Science Division, College of Nursing, University of Arizona, Tucson, AZ, United States

**Keywords:** sleep coaching, sleep apps, CBT-I, insomnia, sleep problems

## Abstract

**Objectives:**

The present study evaluated whether completers of a 12-week app-based, personalized text supported sleep coaching program demonstrated improvements in sleep continuity, sleep duration, and reduced use of sleep aids.

**Methods:**

Data were obtained from Sleep Reset, a 12-week consumer product that offers app-based sleep education and monitoring, along with personalized text-based sleep coaching provided by live coaches. Five hundred sixty-four completers were included in the study. Pre-post changes for sleep latency (SL), wake after sleep onset (WASO), number of awakenings (NWAK), total sleep time (TST), sleep efficiency (SE%) and use of “sleep aids” were evaluated. To evaluate whether the program produced meaningful results, the proportion of participants who demonstrated reductions in SL, WASO, and NWAK, and increases in TST and SE% were examined.

**Results:**

Mean SL was reduced by 11 min, mean WASO was reduced by 28 min, mean SE% increased by 6.6%, and mean TST increased by about 44 min. Of those who reported using “sleep aids” during Week 1, 41% no longer used them by week 12. Those with low SE% at baseline demonstrated greater improvements in SL (16.2 vs. 5.7 min), WASO (47.3 vs. 7.2 min), SE% (11.2 vs. 1.6%), and TST (65.3 vs. 31.2 min). Those with ≤ 6 h of sleep at baseline demonstrated greater improvements in WASO (36.8 vs. 22.3 min), SE% (10.1 vs. 4.3%), and TST (85.1 vs. 25.5 min).

**Conclusion:**

Participants that completed the app-based, personalized text supported coaching intervention reported subjective improvements in sleep duration and quality that suggest more beneficial effects particularly in those with lower sleep efficiency or sleep duration at baseline. An effective sleep coaching program that utilizes trained sleep coaches with access to board-certified providers, may provide a valuable resource for subclinical populations.

## Introduction

Almost half of all Americans say they feel sleepy during the day, with 62% using “shake it off” as their primary way of coping (National Sleep Foundation, 2020). The prevalence of sleep-related problems in the United States population is high. These problems include a wide range of sleep disorders and subclinical sleep difficulties that can be independent of or related to other conditions and can impact quality of life (Lee et al., 2021). To address this, an ecosystem of services is needed, ranging from the most intensive for the most severe problems to more accessible services for the large number of people with subclinical problems. One way to conceptualize this system would be the “stepped care” model, where lower-level care is most accessible to the widest number of people, and as issues become more severe individuals “step up” to higher levels of care (Wong et al., 2021; Muench et al., 2022).

In the case of sleep health, clinical interventions at the higher end of the spectrum such as Cognitive Behavioral Therapy for Insomnia and Positive Airway Pressure therapy for sleep-disordered breathing are well-established (Patil et al., 2019; Muench et al., 2022). For more minor concerns, however, there are few interventions that have been formally evaluated or have empirical support. Examples of less intensive interventions include sleep hygiene education (Chung et al., 2018) and supplements (Chan and Lo, 2022). More recently, sleep coaching programs have emerged. Sleep coaching differs from therapy, in that the goal of coaching is not to diagnose or manage clinical conditions (Holzinger et al., 2019), but rather to provide education, support, and motivation to engage in helpful behaviors and perspectives.

A recent pilot study focused on improving sleep in generally healthy adult volunteers found that a 4-week sleep coaching program comprised of sleep schedule recommendations and targeted SMS messaging led to a statistically significant improvement in overall sleep health in most participants (Schneider et al., 2023). In the same study, it was also noted that higher rates of intervention adherence were correlated with improved overall sleep health at post-intervention.

Sleep coaching has also been shown to be effective in populations experiencing elevated occupational stress due to shift work. In one study, adult shift workers who completed a 2-day sleep improvement seminar demonstrated improved overall sleep quality and daytime sleepiness compared to baseline. Clinically significant improvements were found across several key sleep metrics, including sleep onset latency, diurnal fatigue, and subjective sleep quality (Holzinger et al., 2019). Similarly, a study examining the efficacy of a sleep health coaching intervention to improve sleep in adult members of the German armed forces demonstrated significant improvements in sleep quality, insomnia severity, and daytime sleepiness when compared to a waitlist control group (Danker-Hopfe et al., 2018). Participants in the intervention group also demonstrated significant improvements in sleep onset latency and sleep efficiency as measured by polysomnography (Danker-Hopfe et al., 2018). The results of these studies suggest that sleep coaching has a positive effect on subjective (and objective) sleep quality and daytime sleepiness in adult shift workers with high levels of occupational stress.

Sleep coaching may also have inherent value in adolescent populations dealing with chronic illnesses. A recent pilot study examined the efficacy of a sleep-promoting intervention in adolescents with T1DM. The sleep coaching program was found to be feasible and acceptable, and it increased sleep duration and efficiency for teens in the intervention group (Jaser et al., 2020). On average, sleep duration increased by 48 min. This was especially meaningful because a 15–20-min increase in TST has been previously associated with diabetes treatment adherence in adolescents with T1DM (McDonough et al., 2017).

Taken together, sleep health seems to be amenable to coaching interventions in various non-sleep disordered populations. This is particularly true for interventions that include education and support for beneficial sleep health behaviors. Even among subclinical populations, small changes in population-level sleep health can impact population health. For example, recent work showed that even a few minutes increase in sleep duration over several weeks was associated with a measurable improvement in population-level resting heart rate (Rezaei and Grandner, 2021). Moreover, a few minutes decrease in population habitual sleep duration was associated with increased cardiometabolic risk (Rezaei and Grandner, 2021). Therefore, sleep coaching may represent an opportunity for improving population sleep health for individuals without sleep disorders, allowing sleep-related therapies to be prioritized for those who do have diagnosable sleep conditions. Despite this, there are relatively few studies that systematically evaluate whether sleep coaching interventions in real-world situations might result in measurable improvements in sleep health.

The present study evaluated whether individuals who completed a 12-week app-based, personalized text supported, commercially available sleep coaching program demonstrated improvements in sleep continuity (sleep latency, wake after sleep onset, number of awakenings, sleep efficiency), sleep duration, and use of substances to aid sleep. It was hypothesized that the intervention would result in reduced sleep latency, wake after sleep onset, number of awakenings, and sleep-aid use and increased sleep duration and sleep efficiency. Further, it was hypothesized that these effects would be greater among those who initiated the program with worse sleep continuity (i.e., lower sleep efficiency) and/or shorter sleep duration.

## Methods

### Data source

Data were obtained from Sleep Reset (Simple Habit, Inc.), a commercial product that consists of an online app-based, personalized text supported sleep coaching program. *N* = 564 consecutive individuals who completed the 12-week program were included in the study. The Sleep Reset program is publicly and commercially available and participants paid to use the app and voluntarily joined the program on their own. Before joining the program, potential participants were screened in order to identify the presence of medical or psychological disorders that would be contraindicated for a non-clinical coaching program, as well as likely sleep disorders (including insomnia disorder, sleep apnea, sleep-related movement disorders, parasomnias, and other disorders) via standardized screening questionnaires. For example, participants who indicated that they had a history of bipolar disorder, seizure disorder, any risk of self-harm or harm to others, inability to function due to a mental or physical health crisis, or diagnosed sleep disorder (including insomnia, narcolepsy, sleep apnea, restless legs syndrome, REM sleep behavior disorder, circadian rhythm disorders, or sleep paralysis, or who indicated an exclusionary condition were not enrolled in the program and were referred for outside evaluation and treatment as necessary). Individuals whose reports were not consistent with a medical or psychiatric condition that would preclude participation in a coaching program, or a likely sleep disorder, were enrolled in the program. These individuals consented to have their deidentified data used for research via the terms of service to which they agreed when joining the program.

### Sleep Reset program

**Figure 1:**
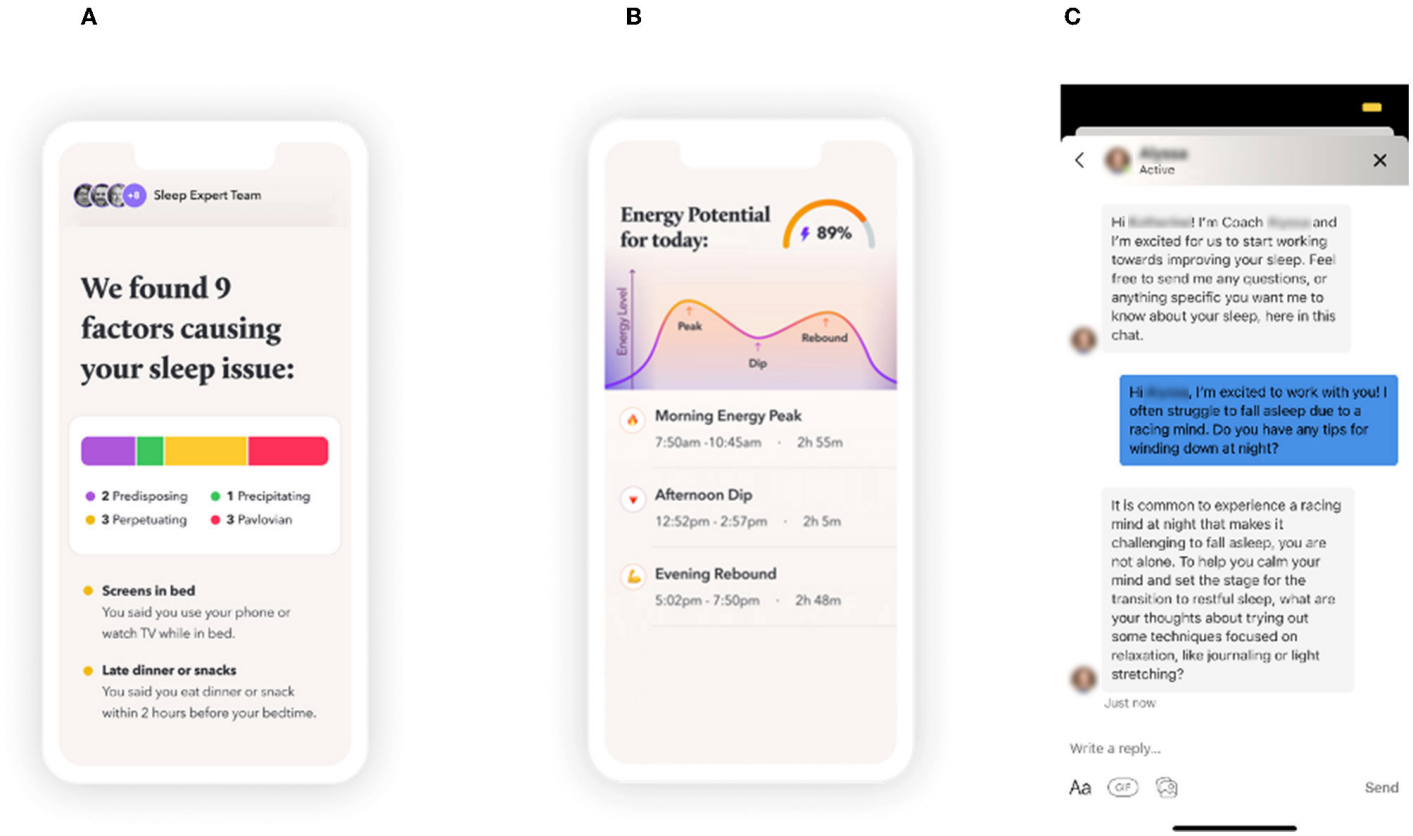
**(A–C)** Sleep reset app. Reproduced with permission from Simple Habit, Inc.

The Sleep Reset program consists of a screening process (to identify individuals who may not be appropriate for coaching and who may need more intensive clinical intervention), an onboarding process (to orient them to the program and set expectations), and then a 12-week intervention period that includes (1) tracking and assessment, (2) education about healthy sleep and circadian habits, and (3) interactive coaching with a live coach. The participants can text their sleep coach directly through the app as needed to ask questions. The sleep coaches use the texts to directly deliver additional recommendations and feedback to the participants. Figure 1 displays screenshots of the Sleep Reset app, populated with sample data (no real information from real participants included, to protect privacy), including (A) a sample feedback page identifying some areas for discussion, (B) a sample feedback page illustrating circadian fluctuations in energy levels, and (C) a sample chat window with a coach.

In addition to the personalized recommendations and feedback from the sleep coaches delivered via app-based text messages, the 12-week program has a standardized curriculum for all participants that includes the following content:

- Week 1: Complete intake & understand the basics: Complete sleep assessment and customize your program; receive your sleep evaluation; introduction to program methodology and key topics: sleep compression, stimulus control circadian rhythm, sleep drive, sleep anxiety; set your first sleep reset schedule (e.g., sleep compression).
- Week 2: Start your sleep reset: Tips to support sleep compression and stimulus control; exercises: rewriting your sleep story, gratitude journaling, worry list, deep breathing, guided imagery.
- Week 3: Support your sleep reset with daytime hygiene: Practices: light exposure, physical movement, caffeine intake, strategies for napping; exercise on self-talk.
- Week 4: Support your sleep reset with evening hygiene, part 1: Bedtime issues: meals and snacking before bed, noise, light, temperature, smell and sleep, sleep supplements; deep breathing exercise.
- Week 5: Support your sleep reset with evening hygiene, part 2: Bedroom issues: sleeping positions, mattresses, other sleep technology; exercise on rewriting your sleep story.
- Week 6: Support your sleep reset with physical activity and nutrition: The connection between sleep and physical movement: rest and recovery, stretching and yoga; sleep-promoting and sleep-disrupting food and drink, alcohol and sleep, intermittent fasting and sleep; the connection between sleep and weight gain/loss.
- Week 7: Normalize setbacks and build confidence with self-compassion: Exercises: free association, worry list exercise, belly breathing exercise; practicing patience; practicing self-compassion.
- Week 8: Normalize setbacks and build confidence with anxiety relief: The relationship between sleep and chronic stress; anxiety and stress dreams; progressive muscle relaxation exercise; self-reflection exercise.
- Week 9: Reflection and habit maintenance: Revisiting key topics from earlier in the program: stimulus control, circadian rhythm, sleep anxiety.
- Week 10: Reflection and habit maintenance: Revisiting key topics from earlier in the program: evening hygiene, daytime hygiene.
- Week 11: Wrap-up and next steps: Reflection on the program; revisiting key exercises from earlier in the program: mindfulness, body scanning, deep breathing, stretching & yoga, progressive muscle relaxation.
- Week 12: Wrap-up and next steps: Rewriting your new sleep story; revisiting key topics from earlier in the program: patience, self-compassion.

Tracking and assessment include questionnaires and a daily sleep diary. This diary is based on the consensus sleep diary (CITE) and assesses sleep continuity with the goal of measuring time in sleep latency (SL), wake after sleep onset (WASO), number of awakenings (NWAK), total sleep time (TST, calculated based on time in bed, subtracting SL and WASO), and sleep efficiency (SE%, calculated as the ratio of TST to total time in bed). In addition, participants were asked daily if they used any “sleep aids” including melatonin or any other substances. Participants indicated yes or no; however, no information about the type or dosage of sleep aids used was recorded.

Since there is no established credential for sleep coaching, a standard training protocol was developed for this program, which included aspects of (1) general sleep and circadian science principles, (2) behavioral sleep and circadian interventions, (3) coaching orientation and techniques, (4) recognizing scope of expertise and identification of situations requiring referral, and (5) motivational interviewing techniques. A written manual that contains much of this information is also made available to all coaches, and all coaches have on-call access and regular contact with a lead coach and licensed clinical psychologist. In addition, Sleep Reset staff meet weekly with a licensed clinical psychologist board-certified in Behavioral Sleep Medicine to address more complex issues as they arise. All coaching interventions are delivered via text-based interactions within the app (see Figure 1).

### Statistical analyses

To evaluate whether the intervention resulted in pre-post changes to sleep parameters, paired samples *t*-tests were conducted for SL, WASO, NWAK, TST, and SE%. To evaluate use vs. non-use of “sleep aids,” a 2 × 2 chi-square test was conducted, with a *post-hoc t*-test to examine whether individuals changed their use of sleep aids from week 1 to 12. Secondary analyses compared the magnitude of pre-post differences across two sets of *post-hoc* groups, again using *t*-tests. First, differences were examined between those who reported low sleep efficiency at baseline (< 85%) compared to those who reported higher sleep efficiency (≥85%). Second, differences were examined between those who reported short sleep at baseline (6 h or fewer), compared to longer sleepers who reported more than 6 h of habitual sleep at baseline. In addition, age and gender-adjusted regression analyses examined whether observed group differences in pre-post effects were maintained after demographic adjustments. To evaluate whether the program produced clinically meaningful results, the proportion of participants who demonstrated reductions in SL, WASO, and NWAK, and increases in TST and SE%, were calculated. A priori, changes evaluated in the complete sample were defined as ≥10 min, ≥ 30 min, and ≥ 50% reduction in SL, ≥10 min, ≥30 min, and ≥60 min reduction in WASO, ≥1 or ≥2 fewer awakenings, ≥5% or ≥10% gain in sleep efficiency, and ≥15, ≥30, ≥60, ≥90, and ≥120 min gained in TST. Then, age and gender-adjusted regression analyses examined whether these improvements were more likely to be seen in short sleepers and/or those with low sleep efficiency at baseline. Additional secondary analyses computed the percentage of participants who crossed thresholds for SL (from ≥30 to < 30 min), WASO (from ≥30 to < 30 min), NWAK (from >3 awakenings to ≤ 3 awakenings), SE% (from < 85 to ≥85%), and TST (from ≤ 360 to ≥390 min). All statistical analyses were computed using STATA 17.0 (STATACORP, College Station, TX).

## Results

### Characteristics of the sample

The sample consisted of *N* = 564 adults. Of these, demographics data were available for *N* = 523. Among these, 65.2% were female, 34.4% were male, and 0.4% were intersex. Regarding age distributions, 9.2% were 18–29, 19.1% were 30–39, 22.4% were 40–49, 20.5% were 50–59, 18.2% were 60–69, and 10.7% were 70 or older. Participants were recruited through direct advertising and word of mouth.

### Pre-post changes in sleep parameters

Self-reported SL, WASO, SE%, NWAK, and TST are presented in Table 1 for Week 1 and Week 12. Mean SL was reduced by ~11 min, whereas mean WASO was reduced by ~28 min. In addition, SE% increased by an average of 6.6%, NWAK reduced by 0.4 awakenings per night, and TST increased by about 44 min. All differences were statistically significant at the *p* < 0.00001 level.

**Table 1:** Pre-post differences in sleep characteristics of program participants (*N* = 564).

	**Week 1**			**Week 12**			***t*** **-test**	
	**Mean**	**SD**	**Range**	**Mean**	**SD**	**Range**	***t***	***p*-value**
Sleep latency (min)	27.52	21.52	2–135	16.50	16.32	2–120	−12.40	−6.66 × 10^−16^
Wake after sleep onset (min)	81.00	45.96	10–253	53.02	39.55	5–247	−14.42	5.50 × 10^−17^
Sleep efficiency (%)	82.21%	10.11%	38%−98%	88.79%	8.40%	36%−99%	15.49	−4.44 × 10^−16^
Number of awakenings (#)	2.38	0.83	1–5	1.97	0.78	1–5	−9.61	5.55 × 10^−16^
Total sleep time (min)	383.27	82.56	139–720	427.32	77.16	158–720	11.21	−4.44 × 10^−16^

When participants were split into groups representing low baseline SE% vs. high, those with low SE% at baseline demonstrated greater improvements overall. These results are displayed in Table 2. Overall, changes were greater for SL (5.7 vs. 16.2 min), WASO (7.2 vs. 47.3 min), SE% (1.6 vs. 11.2%), and TST (31.2 vs. 65.3 min) in those with low baseline SE%. Regression analyses adjusting for gender and age group found that those with low baseline SE% decreased their SL by an additional 10.80 min [95% CI: (−14.41, −7.19), *p* < 0.0005], decreased their WASO by an additional 40.21 min [95% CI: (−47.36, −33.06), *p* < 0.0005], increased their sleep efficiency by an additional 9.49% [95% CI: (7.96%, 11.02%), *p* < 0.0005], and increased their sleep duration by an additional 31.61 min [95% CI: (13.64, 49.58), *p* < 0.0005].

**Table 2:** Differences in pre-post change in sleep parameters according to baseline sleep efficiency.

	**Low sleep efficiency (*****N*** = **292)**		**High sleep efficiency (*****N*** = **272)**		***t*** **-test**	
	**Change**	**95% CI**	**Change**	**95% CI**	***t***	***p*-value**
Sleep latency (min)	−16.17	(−18.94, −13.40)	−5.65	(−7.58, −3.72)	6.06	2.49 × 10^−09^
Wake after sleep onset (min)	−47.32	(−52.94, −41.71)	−7.23	(−11.04, −3.41)	11.46	4.44 × 10^−16^
Sleep efficiency (%)	11.20%	(10.01%, 12.38%)	1.63%	(0.78%, 2.48%)	−12.76	3.33 × 10^−16^
Number of awakenings (#)	−0.48	(−0.60, −0.36)	−0.34	(−0.45. −0.22)	1.62	0.106
Total sleep time (min)	65.32	(53.69, 76.95)	31.19	(18.83, 45.54)	−3.96	8.45 × 10^−5^

When participants were split into groups representing short baseline TST vs. longer sleep, those with shorter sleep duration at baseline also demonstrated greater improvements overall. These results are displayed in Table 3. Overall, changes were greater for WASO (22.3 vs. 36.8 min), SE% (4.3 vs. 10.1%), and TST (25.5 vs. 85.1 min) in those with baseline shorter TST. Regression analyses adjusting for gender and age group found that short sleepers decreased their WASO by an additional 15.98 min [95% CI: (−24.17, −7.80), *p* < 0.0005], increased their SE% by an additional 6.02% [95% CI: (4.29%, 7.75%), *p* < 0.0005], and increased their sleep duration by an additional 54.48 min [95% CI: (36.11, 72.85), *p* < 0.0005].

**Table 3:** Differences in pre-post change in sleep parameters according to baseline sleep duration.

	**Short sleep (*****N*** = **221)**		**Longer sleep (*****N*** = **343)**		***t*** **-test**	
	**Change**	**95% CI**	**Change**	**95% CI**	***t***	***p*-value**
Sleep latency (min)	−12.8	(−15.79, −9.81)	−10	(−12.15, −7.84)	1.53	0.126
Wake after sleep onset (min)	−36.82	(−43.48, −30.17)	−22.29	(−26.79, −17.80)	3.70	0.0002
Sleep efficiency (%)	10.14%	(8.58%, 11.70%)	4.29%	(3.44%, 5.15%)	−6.99	< 0.0001
Number of awakenings (#)	−0.49	(−0.63, −0.35)	−0.36	(−0.46, −0.25)	1.58	0.115
Total sleep time (min)	85.10	(40.30, 97.18)	25.51	(14.41, 36.60)	−6.95	1.01 × 10^−11^

**Figure 2:**
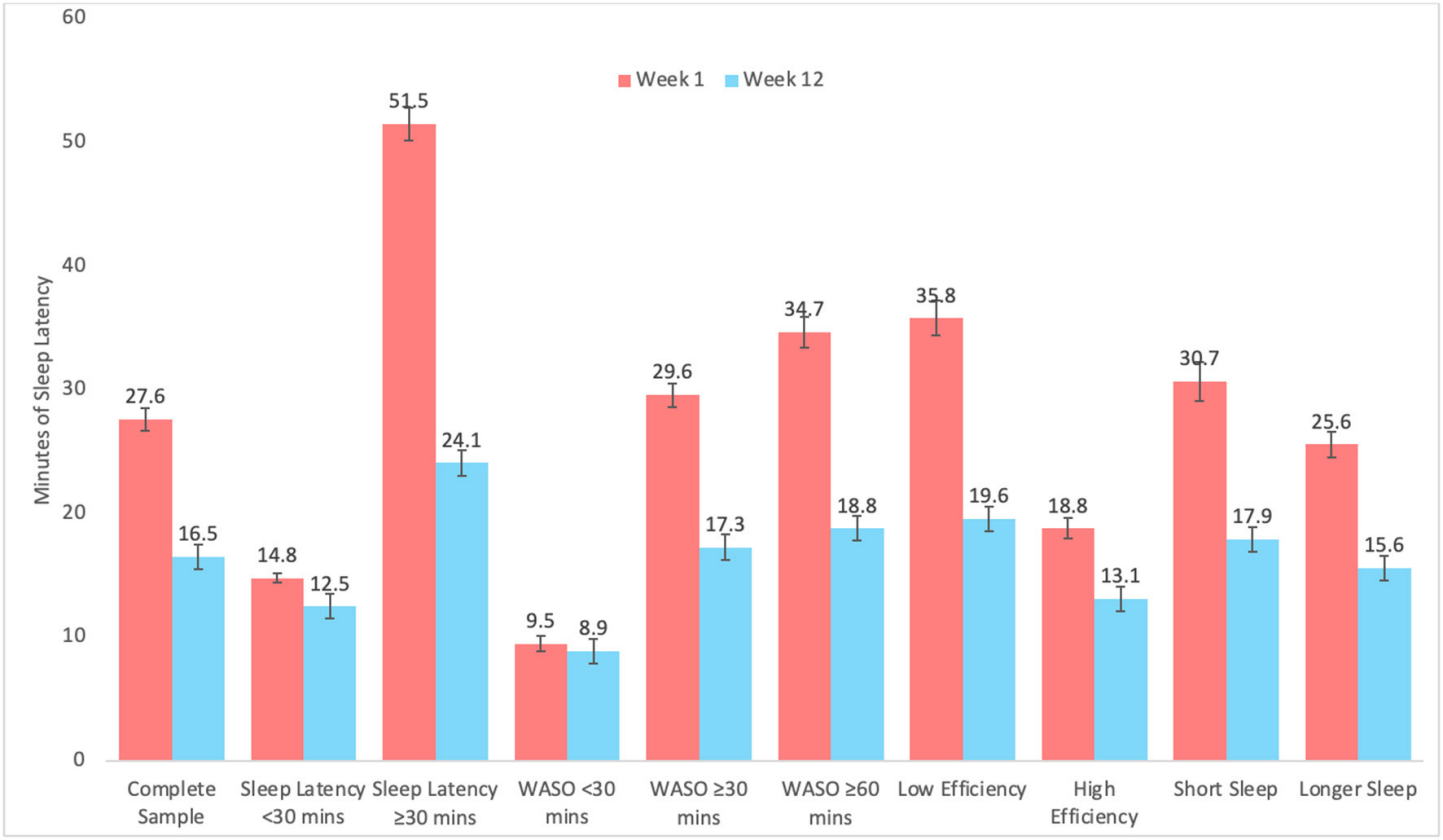
Mean and standard error of sleep latency (SL) at week 1 and week 12 for the complete sample, as well as study sub-groups.

**Figure 3:**
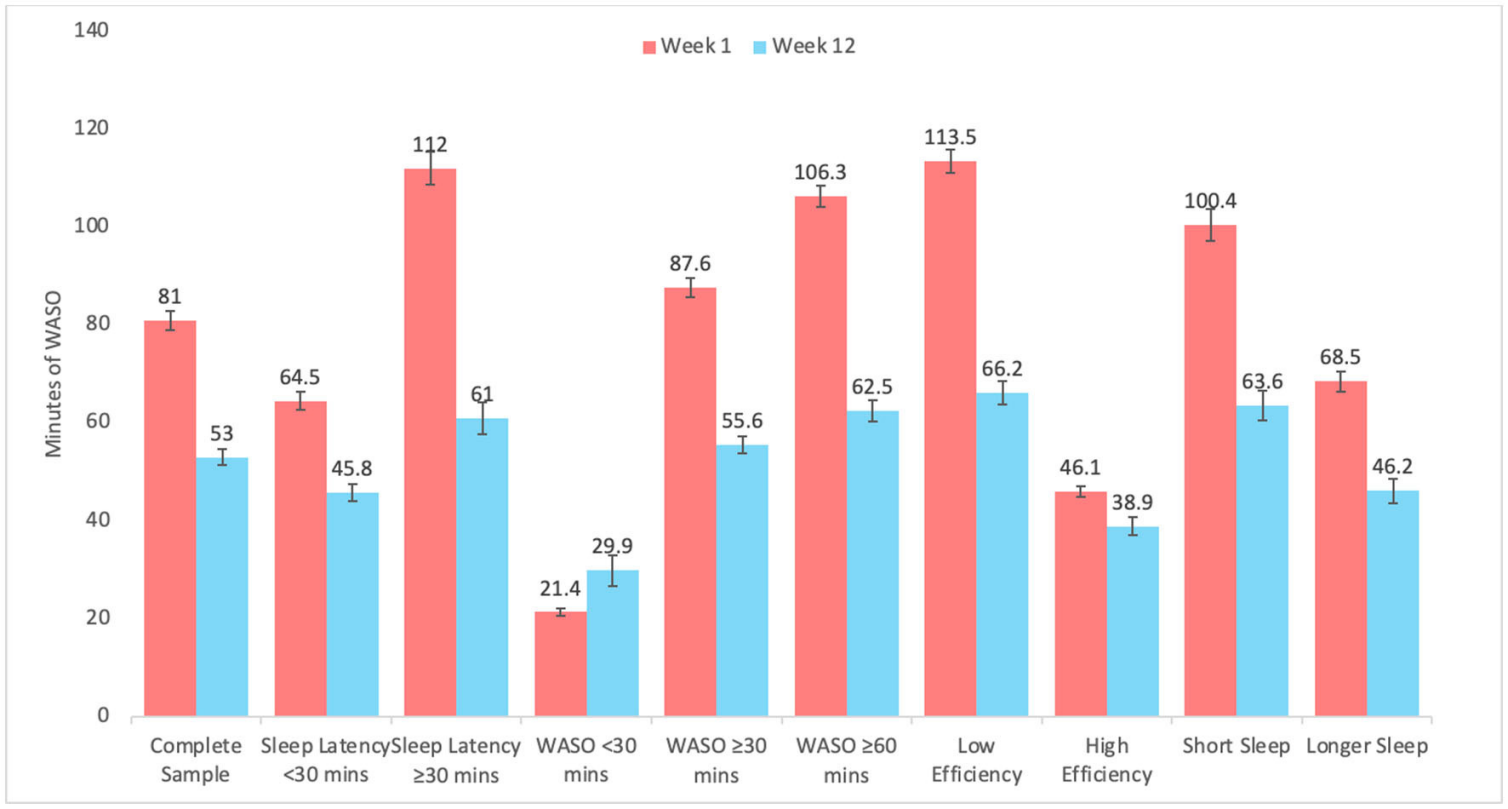
Mean and standard error of wake after sleep onset (WASO) at week 1 and week 12 for the complete sample, as well as study sub-groups.

**Figure 4:**
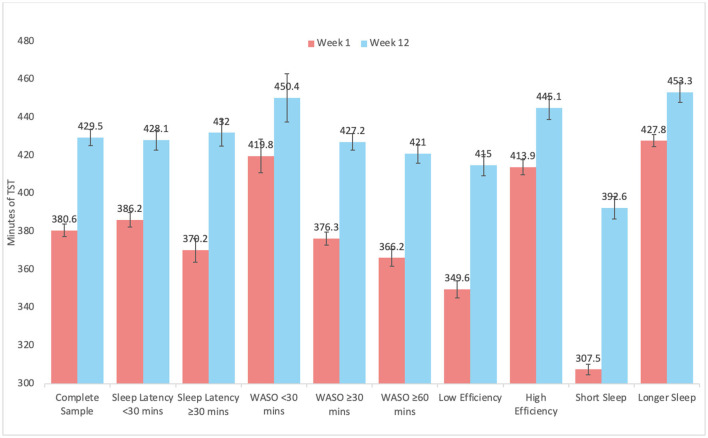
Mean and standard error of total sleep time at week 1 and week 12 for the complete sample, as well as study sub-groups.

Graphical representation of changes by subgroups are depicted in Figure 2 for SL, Figure 3 for WASO, and Figure 4 for TST. In each case, the figures depict the mean and standard error for values at Week 1 and Week 12 for the complete sample, those with SL < 30 min, SL ≥30 min, WASO < 30 min, WASO ≥30 min, WASO ≥60 min, < 85% SE%, ≥85% SE%, ≤ 6 h TST, and >6 h TST, in order to visualize changes relative to baseline values. As an exploratory analysis, paired *t*-tests (with familywise Bonferonni-corrected criterion of *p* < 0.005 per group of analyses) showed that all pairwise comparisons were statistically significant, except that those who began the program with low WASO did not show statistically significant change in SL, WASO, or TST.

### Changes to use of sleep aids

Among participants, 54.96% reported using sleep aids at least once during the first week of participation. This was reduced to 40.6% by week 12. Among those that did not use sleep aids at baseline, 81.89% continued to avoid them at week 12 while 18.11% had begun using them during the study period. Among those who used sleep aids at baseline, 59.03% were still using them at week 12 while 40.97% had stopped. A chi-square test was statistically significant [*X*^2^(1) = 96.9, *p* = −8.88 × 10^−16^], and a *post-hoc t*-test showed that sleep aid use was reduced overall pre-post [*t*_(563)_ = −6.37, *p* = 3.93 × 10^−10^].

### Threshold changes

The proportion of participants that showed different amounts of change to each variable are reported in Table 4. Irrespective of baseline values, 43% decreased SL by at least 10 min, 16% reduced SL by at least 30 min, and 44% reduced SL by at least 50% from baseline. Regarding WASO, 66% reduced WASO by at least 10 min, 45% reduced WASO by at least 30 min, 17% reduced WASO by at least 60 min. Regarding NWAK, 45% reported at least 1 fewer awakening and 13% reported at least 2 fewer awakenings. Regarding SE%, 54% showed at least 5% greater SE% and 32% showed at least 10% higher SE%. Regarding TST, 68% slept at least 15 min more, 59% slept at least 30 min more, 36% gained at least 60 min, 21% gained at least 90 min, and 12% gained at least 2 h of TST.

**Table 4:** Participants experiencing change in sleep-related variables.

**Variable**	**Change**	**%**	**Short sleepers vs. others**			**Low sleep efficiency vs. others**		
			**OR**	**95% CI**	***p*** **-value**	**OR**	**95% CI**	***p*** **-value**
SL	≥10 min reduction	43.09%	1.43	1.00, 2.05	0.051	2.84	1.98, 4.07	< 0.0005
	≥30 min reduction	15.96%	1.31	0.81, 2.12	0.267	3.38	1.99, 5.74	< 0.0005
	≥50% reduction	43.97%	1.38	0.97, 1.98	0.077	1.80	1.27, 2.55	0.001
WASO	≥10 min reduction	65.78%	1.56	1.07, 2.29	0.022	3.75	2.55, 5.51	< 0.0005
	≥30 min reduction	44.86%	2.28	1.58, 3.27	< 0.0005	7.34	4.96, 10.88	< 0.0005
	≥60 min reduction	17.31%	2.76	1.72, 4.43	< 0.0005	23.33	9.27, 58.70	< 0.0005
NWAK	≥1 fewer awakening	44.68%	1.02	0.71, 1.46	0.902	1.13	0.80, 1.60	0.477
	≥2 fewer awakenings	12.94%	1.76	1.04, 2.97	0.034	1.76	1.04. 3.00	0.036
SE%	≥5% gain	54.43%	2.99	2.05, 4.35	< 0.0005	5.74	3.93, 8.40	< 0.0005
	≥10% gain	31.56%	3.97	2.68, 5.90	< 0.0005	19.47	11.06, 34.29	< 0.0005
TST	≥15 min gained	68.44%	5.59	3.49, 8.95	< 0.0005	2.31	1.58, 3.38	< 0.0005
	≥30 min gained	59.04%	5.22	3.45, 7.90	< 0.0005	2.10	1.47, 2.99	< 0.0005
	≥60 min gained	36.17%	5.33	3.60, 7.90	< 0.0005	3.51	2.39, 5.16	< 0.0005
	≥90 min gained	21.10%	5.86	3.66, 9.40	< 0.0005	4.88	2.96, 8.04	< 0.0005
	≥120 min gained	12.23%	6.96	3.70, 13.07	< 0.0005	6.38	3.16, 12.86	< 0.0005

When these gains were compared in adjusted analyses between short sleepers vs. others (reported in Table 4), short sleepers were 56% more likely to reduce WASO by at least 10 min, 128% more likely to reduce WASO by at least 30 min, and 176% more likely to reduce WASO by at least 60 min. They were also 76% more likely to reduce number of awakenings by at least 2, 199% more likely to experience a gain in SE% of at least 5%, and 297% more likely to gain at least 10% in SE%. Regarding change in TST, short sleepers were 459% more likely to gain at least 15 min, 422% more likely to gain at least 30 min, 433% more likely to gain at least 60 min, 486% more likely to gain at least 90 min, and 596% more likely to gain at least 2 h of TST.

When these gains were compared in adjusted analyses between those with baseline low sleep efficiency vs. others (reported in Table 4), those with low baseline sleep efficiency were 184% more likely to reduce SL by at least 10 min, 238% more likely to reduce SL by at least 30 min, and 80% more likely to report a reduction in SL by at least 50% of baseline. They were also 275% more likely to reduce WASO by at least 10 min, 634% more likely to reduce WASO by at least 30 min, and 2,233% more likely to reduce WASO by at least 60 min.

When evaluating the percentage of those who crossed a threshold, of the *N* = 196 who had a baseline SL of at least 30 min, mean reduction in sleep latency was 27.4 min, or 53%; 71.94% of those had a SL of < 30 min at week 12. Of the *N* = 508 participants who reported WASO of at least 30 min at baseline, 30.91% of those had a WASO of < 30 min at week 12. Of those that had a WASO over 60 min, WASO decreased by a mean of 43.8 min, or 41.2%. Of the *N* = 47 participants who reported more than 3 awakenings at baseline, 63.83% reported 3 or fewer by week 12. Of the *N* = 292 participants who had a SE% below 85% at baseline, 64.04% had a SE% of at least 85% by week 12. Of the *N* = 221 participants who started the program with a TST of ≤ 6 h, 49.77% achieved at least 6.5 h by week 12, and 24.43% achieved at least 7 h. In addition, 91.84% of the sample reported that, at baseline, they experienced at least one of the following: SL or WASO **≥**30 min, NWAK >3, SE% < 85%, and/or TST ≤ 6 h. Of those, 96.76% reported at least one of the following: SL or WASO reduction of at least 10 min, NWAK reduction of at least 1 awakening, SE% increase of at least 5%, and/or TST increase of at least 15 min. Further, 77.80% of individuals with at least one of the problems noted reported at least one of the following: SL or WASO reduction or TST increase of at least 30 min, SE% increase of at least 10% and/or NWAK reduction by at least 2 awakenings per night.

## Discussion

The present study evaluated the uncontrolled effects of a 12-week app-based, personalized text supported digital sleep coaching program. Overall, the program resulted in relatively robust participant reported improvements in sleep duration and continuity, especially among those with lower sleep efficiency and shorter sleep duration at baseline. Use of sleep aids was also reduced.

The magnitude of effects seen in this intervention are larger than those generally seen in sleep coaching interventions. It should be noted; however, that this analysis focused on completers and that the intervention was relatively intensive (12 weeks). Future analyses that examine this intervention vs. a control group, using intent-to-treat analyses, may show reduced effects. It is possible that this intervention's strengths contributed to its observed effectiveness. First, the focus on evidence-based behavioral sleep medicine principles may have contributed. Previous research shows that even basic behavioral sleep education can result in measurable improvements to sleep health (Chung et al., 2018; Vitale et al., 2019; Philippens et al., 2022); expansion of this to include evidence-based interventions (e.g., circadian and stimulus control approaches) may have further improved efficacy (Kansagara, 2016; Edinger, 2021). Second, the personalized approach of the coaches may have allowed the intervention to incorporate a degree of flexibility and personalization that is typically lacking in app-delivered interventions (Kalmbach et al., 2022; Liang et al., 2022; Kalmbach and Cheng, 2023).

One notable finding from this study was a statistically and clinically significant increase in TST that was accompanied by an increase in SE. Typically, nominal increases in TIB that produce increases in TST generally produce decreases in SE, owing to a reduced sleep pressure distributed over a greater amount of time (Levine, 1988). On the contrary, when individuals have high homeostatic sleep pressure, and TIB is increased, this can result in increased TST that accompanies an increase in SE, such as in CBT-I. One example would be the upward titration of TIB following sleep restriction following CBT-I (Miller, 2014). Another example would be sleep recovery opportunity following sleep deprivation (Jay, 2007). The present study suggests the increase in TST accompanied by an increase in SE represents a scenario where participants increased both TST and SE, which subsequently indicates that they increased TIB less than they increased TST.

When results were stratified according to various user groups, notable limits to the program were observed. For example, individuals with short sleep latency did not show improvements in sleep latency, those with little WASO and high sleep efficiency did not show improvements in sleep latency or WASO, and those with low WASO, high sleep efficiency, and longer sleep duration increased their sleep duration by the lowest amount. All of these findings are reasonable and should be expected—those with relatively little room for improvement did not show much improvement. In addition to supporting the validity of the data, these results suggest that benefits from the program were seen in a wide range of users, but some users should not expect much change in these parameters.

These findings are promising and may substantially contribute to a stepped approach to sleep care. While it is estimated that 10%−15% of Americans have chronic insomnia (Ram, 2010), 35.3% report getting < 7 h of sleep a night (CDC, 2009). Both disordered sleep and short sleep are considered a part of the public health crisis in the United States (Colten et al., 2006). However, efforts primarily focus on intervening with disordered sleep, thus neglecting a significant portion of impacted individuals. Daytime sleepiness as a result of short sleep results in more accidents (Philip et al., 2014; Chattu et al., 2018), poorer health (Ram, 2010; Chattu et al., 2018), and poorer productivity (Hafner et al., 2017). Fortunately, coaching paradigms are well-suited to address issues with short sleep with minimal burden on people and providers. The app provides recommendations to curb unhealthy sleep behaviors before they become entrenched and problematic. Insomnia is a behavioral disorder in which maladaptive behaviors such as napping, engaging in activities in bed, and maintaining an inconsistent sleep schedule maintain sleep challenges (Spielman et al., 1987). Many of these behaviors are perpetuated because people consider them “common sense” (e.g., taking a nap after a poor night of sleep to “catch up”) or people do not see the connection between their behaviors and their sleep (e.g., eating in bed). By educating and encouraging people before the development of insomnia, we may prevent it entirely. Importantly, the app does not require the involvement of a medical provider. Thus, an already bloated healthcare system will not be further burdened by administering a clinical intervention to subclinical patients.

### Limitations

The main limitation of this study is a lack of a control group. It is possible that simply engaging with the program (or regression to the mean) explains these differences. However, the magnitude of change makes this unlikely, although future work will need to compare this type of intervention to a control group to clarify the effects of the intervention. Another major limitation of this study is that it did not perform an intent-to-treat analysis; rather, inclusion in analyses required completion of the 12-week program. This decision was made for two reasons: first, since this is a commercially-available product, many people may sign up out of curiosity or without any real commitment to engage with the program and therefore an intent-to-treat analysis may inadvertently include individuals who actually did not have an intention to engage with the intervention; Second, since this is the first proof-of-concept evaluation of this program, an analysis of completers will provide information as to whether the intervention can, in principle, produce change in parameters of interest.

In addition to these major limitations, reliance on sleep diary data precludes assessment of objective sleep parameters. Further, the intervention contained several components, including coaching, education, feedback, tracking, etc. It is possible that some of these elements were more important than others in the implementation of the intervention. Future studies should include a dismantling approach to examine elements of the intervention separately. Also, screening procedures aimed to exclude individuals who are not appropriate for sleep coaching, including those with likely sleep disorders. While baseline data suggest that many individuals still had sleep difficulties, this may have resulted in ceiling effects, especially for those who did not start the program with lower sleep efficiency or shorter sleep duration.

## Conclusion

In this pilot evaluation of a novel, app-based, personalized text-supported sleep coaching intervention, completion of the 12-week program was associated with substantial participant reported improvements in overall changes in the sleep parameters of sleep latency, wake after sleep onset, sleep efficiency, sleep duration, and reduced use of sleep-aids in those who reported use at baseline. The participant-reported improvements were greater among those who reported lower sleep efficiency and/or shorter sleep duration at baseline. It is important to again note that there were multiple limitations in the dataset and protocol that limit the generalizability of the findings. Future research should evaluate this intervention in comparison with a control group using intent-to-treat analyses. Implementation of this type of intervention should be evaluated as a viable strategy for improving population sleep health, especially among individuals who have sleep complaints but do not meet diagnostic criteria for sleep disorders.

## Data availability statement

The raw data supporting the conclusions of this article will be made available by the authors, without undue reservation.

## Ethics statement

Ethical review and approval was not required for the study of human participants in accordance with the local legislation and institutional requirements. The participants consented to have their deidentified data used for research via the terms of service to which they agreed when joining the program, and voluntarily joined the program on their own.

## Author contributions

SG was responsible for writing, compiling, completing final edits, and submission. RC and MG were responsible for writing and editing. RF was responsible for editing and contributing. All authors contributed to the article and approved the submitted version.
